# Genetic Risk, Inflammation, and Therapeutics: An Editorial Overview of Recent Advances in Aging Brains and Neurodegeneration

**DOI:** 10.14336/AD.2024.0986

**Published:** 2024-10-01

**Authors:** Vivek Gupta, Nitin Chitranshi, Veer Bala Gupta

**Affiliations:** ^1^Macquarie Medical School, Faculty of Medicine, Health and Human Sciences, Macquarie University, North Ryde, Sydney, NSW 2109, Australia.; ^2^School of Medicine, Deakin University, Geelong, VIC 3216, Australia.

**Keywords:** Aging, Neurodegeneration, Neuroprotection, Neuroinflammation, Oxidative stress, Mitochondria

## Abstract

Neurodegenerative disorders, including Dementia, Parkinson's disease, various Vision disorders, Multiple sclerosis, and transsynaptic degenerative changes represent a significant challenge in aging populations. This editorial synthesizes and discusses recent advancements in understanding the genetic and environmental factors contributing to these diseases. Central to these advancements is the role of neuroinflammation and oxidative stress, which exacerbate neuronal damage and accelerate disease progression. Emerging research underscores the significance of mitochondrial dysfunction and protein aggregation in neurodegenerative pathology, highlighting shared mechanisms across various disorders. Innovative therapeutic strategies, including gene therapy, CRISPR-Cas technology, and the use of naturally occurring antioxidant molecules, are being investigated to target and manage these conditions. Additionally, lifestyle interventions such as exercise and healthy diet have shown promise in enhancing brain plasticity and reducing neuroinflammation. Advances in neuroimaging and biomarker discovery are necessary to improve early diagnosis, while clinical and preclinical studies are essential for the translation of these novel treatments. This edition aims to bridge the gap between molecular mechanisms and therapeutic applications, offering insights into potential interventions to mitigate the impact of neurodegenerative diseases. By establishing a deeper understanding of these complex processes, we aim to move closer to effective prevention and treatment strategies, ultimately improving the quality of life for those affected by neurodegenerative disorders.

The aging process is a well-documented risk factor for numerous neurodegenerative disorders. These disorders, which include Alzheimer's disease (AD), Parkinson's disease (PD), multiple sclerosis (MS), amyotrophic lateral sclerosis (ALS), and various dementias, share a common underlying theme of neurodegeneration. These editorial aims to highlight the recent advancements in understanding the molecular mechanisms driving these disorders and the therapeutic strategies being explored to combat them.

## Molecular Mechanisms of Neurodegeneration

Neurodegenerative diseases involve a complex interplay of genetic and environmental factors. With advancing age, several molecular changes occur in the brain and other interacting physiological systems that alter brain homeostasis and predispose individuals to neurodegenerative diseases. One key aspect is the process of neuroinflammation, or "neuroinflammaging," characterized by a chronic, low-grade inflammation that contributes to brain aging. This process has been implicated in the pathogenesis of many age-related neurodegenerative disorders, including AD and PD. Neuroinflammaging involves the upregulation of inflammatory responses, which can exacerbate neuronal damage and contribute to disease progression [1]. Research has also shown that inflammation is a significant factor in the progression of glaucoma, affecting the retinal ganglion cells and optic nerve and leading to vision loss [2, 3].

Oxidative stress is another critical factor in neurodegeneration. The accumulation of reactive oxygen species (ROS) can damage cellular components, leading to death of neurons and impairment in the functions of other supporting cells. For instance, in PD, oxidative stress is a significant contributor to the loss of dopaminergic neurons in the substantia nigra. Similarly, in AD, oxidative damage plays a role in the formation of tau neurofibrillary tangles and amyloid plaques, which are hallmarks of the disease [4]. Recent studies have also highlighted the detrimental effects of oxidative stress on retinal cells, linking AD to retinal degeneration and emphasizing the need for targeted retinal delivery of therapeutic agents [5].

Mitochondrial dysfunction is also a prominent feature of neurodegenerative diseases. Mitochondria, being the powerhouses of the cell, are crucial for neuronal survival and function. However, with aging, mitochondrial efficiency declines, leading to impaired energy production and increased production of ROS. This mitochondrial dysfunction is evident in diseases like AD and PD, where impaired mitophagy which involves the selective degradation of mitochondria by autophagy, contributes to the accumulation of damaged mitochondria and subsequent neuronal death [6].

### Shared Pathological Traits Across Neurodegenerative Diseases

Recent research has identified several shared pathological traits across different neurodegenerative diseases. These include the aggregation of misfolded proteins, such as amyloid-β aggregates in AD, α-synuclein in PD, and huntingtin protein in Huntington's disease (HD) [7]. These protein aggregates disrupt normal cellular function, increase endoplasmic reticulum (ER) stress and trigger a cascade of neurotoxic events.

The recent studies indicate that the effects of neurodegeneration extend beyond the central nervous system, impacting sensory systems and peripheral nerves, emphasizing the systemic nature of neurodegenerative processes. The papers published in this special edition link them to ocular health and highlight the intricate relationship between different neural pathways [8, 9]. Specifically, the impact of AD on the retina has been a focal point, revealing how neurodegenerative processes affect vision and highlighting the potential for retinal biomarkers in diagnosing neurodegenerative diseases [5, 10].

### Neuroprotective Strategies

The quest for effective neuroprotective strategies has led to the exploration of various mechanism-based approaches, ranging from lifestyle interventions to advanced pharmacological treatments. Exercise, in particular, has been shown to have neuroprotective potential by enhancing brain plasticity, reducing inflammation, and improving mitochondrial function, thereby mitigating the effects of aging on the brain and delaying the onset of neurodegenerative diseases [11]. Emerging research has identified phosphodiesterase 5 (PDE5) inhibitors as promising candidates for treating neurocognitive disorders due to their poly-pharmacological potential. These inhibitors enhance cognitive function and neuroprotection by targeting multiple pathological processes, including amyloid deposition, tau phosphorylation, and oxidative stress [12].

Pharmacological interventions are also being actively pursued. Compounds targeting oxidative stress, such as naturally existing antioxidants, have been investigated for their potential to reduce neuronal damage. For example, polyphenolic phytochemicals like phloridzin have been shown to reduce intracellular ROS levels and promote autophagy, thereby extending lifespan and improving disease phenotypes in animal models [13].

In addition, gene therapy and CRISPR-based techniques are being investigated to specifically target genetic defects associated with neurodegenerative diseases. For instance, gene silencing approaches targeting mutant genes in Huntington’s disease (HD) have shown potential in preclinical models [14]. Similarly, CRISPR technology has been utilized to correct genetic mutations in models of ALS and PD [15, 16].

### Clinical and Preclinical Investigations

The utilization of animal models and *in vitro* cell culture systems has been instrumental in advancing our understanding of neurodegenerative diseases. These models allow the scientific community to dissect the intricate mechanisms underlying disease progression and test potential therapeutic strategies. For example, transgenic mouse models of AD and PD have provided valuable insights into the role of amyloid-β and α-synuclein in disease pathology and helped us identify strategies to target these diseases. Recent advancements in cell and gene therapy have spotlighted mesenchymal stem cell-derived extracellular vesicles (MSC-EVs) as a promising cell-free therapeutic option for Alzheimer's disease and other disorders. These MSC-EVs offer low immunogenicity and high safety, with potential to be optimized through gene editing and other modifications for enhanced therapeutic efficacy [17].

Clinical studies are equally important in translating these findings into viable treatments for patients. Advances in neuroimaging and biomarker discovery have enhanced our ability to diagnose neurodegenerative diseases at early stages, allowing for timely intervention. Moreover, clinical trials testing novel therapies, including monoclonal antibodies and small pharmacological molecules, are in various stages of their development and hold promise for the future of neurodegenerative disease treatment. The specific delivery of these molecules to the target brain or retinal cells in a minimally invasive manner however remains a key challenge in the field [18].

Recent research has highlighted the interplay between neurological diseases such as Alzheimer's disease and multiple sclerosis genetic risk factors, viral susceptibility, and inflammation. The study by Cao et al. (2023), identifies AD candidate genes that are susceptible to alterations caused by viral infection and inflammatory responses, offering potential therapeutic targets for both AD and related infectious diseases [19]. Recent studies have also revealed significant sex differences in brain volume and cerebral blood flow (CBF) with aging. Specifically, it is suggested that women tend to have greater total intracranial CBF and cortical gray matter volume, which may contribute to the observed differences in the incidence and progression of neurodegenerative diseases [20].

New research has highlighted the association between choroid plexus (CP) integrity and plasma biomarkers of Alzheimer's disease and neuroinflammation. These findings by Resnick and Egan (2024) suggest that CP structural degradation and resultant plasma biomarker alterations in AD pathology and neuroinflammation, are linked to CP integrity in aging, making it a potential target for early disease detection and treatment monitoring [21]. Similarly, the studies on RNA-binding proteins FUS and TDP-43 have shed light on their roles in amyotrophic lateral sclerosis (ALS) and frontotemporal degeneration (FTD). These proteins undergo liquid-liquid phase separation, forming assemblies that can potentially transform into pathological amyloid fibrils, highlighting potential targets for therapeutic intervention in neurodegenerative diseases [22]. Furthermore, the findings by Di Luca et al. (2021) have revealed that Type 2 diabetes mellitus (T2DM) is involved in disrupting hippocampal neural oscillations and impairs synchrony between the hippocampus and cortex. These alterations, coupled with reduced neurogenesis, may accelerate cognitive impairment in aging individuals with T2DM [23].

### Policy Implications in Public Health

The increasing aging population and prevalence of neurodegenerative diseases in aging populations necessitates an integrated public health strategy that encompasses enhanced screening and early intervention, educational and awareness campaigns, and robust support for caregivers and community health services [24]. These public health policies should focus on enhancing the outreach of widespread screening programs that incorporate genetic testing and biomarker analysis to identify individuals at high risk early on, while also increasing public and professional awareness about key modifiable risk factors such as diet and exercise [25]. Additionally, there is a critical need for expanded resources for caregiver support and community health services to help manage the broader social impacts of neurodegenerative diseases. Funding for ongoing research with long term vision and a cooperative regulatory framework are essential to foster innovation in therapies, including emerging treatments like gene therapy and cell-free therapies like MSC-EVs [26]. These efforts should be supported by clear regulatory pathways to ensure new therapies are brought to market safely and efficiently. Overall, proactive and tailored public health policies are vital to enhance disease prevention, facilitate early diagnosis, and improve treatment outcomes, thereby reducing the overall healthcare burden and enhancing the quality of life for affected individuals and their families [27].

### Conclusions

The papers in this special edition collectively advance our understanding of the complex interplay between aging and neurodegenerative diseases. Understanding these mechanisms is crucial for developing effective neuroprotective strategies. By elucidating the shared molecular mechanisms, genetic predispositions, and potential therapeutic interventions, these studies pave the way for innovative approaches to diagnose, treat, and ultimately prevent neurodegenerative disorders. This edition serves as a testament to the collaborative efforts of researchers worldwide, dedicated to unravelling the mysteries of aging and several neurodegenerative conditions that are associated with aging. The special edition elucidates novel diagnostic and therapeutic strategies and provides insights from both clinical and preclinical investigations to improve the quality of life for affected individuals. Together, these efforts will contribute to a comprehensive understanding of neurodegenerative diseases and the development of effective interventions to combat their debilitating effects. We invite scientific community, researchers and students to delve into these high-quality original research articles and comprehensive reviews, which offer valuable insights into the pathological mechanisms of neurodegenerative disorders and aging and explore the potential of diverse neuroprotective strategies. Together, these contributions will drive forward the field of neurodegeneration research and contribute to the development of effective interventions for these debilitating conditions.
